# The Effect of Polyol Composition on the Structural and Magnetic Properties of Magnetite Nanoparticles for Magnetic Particle Hyperthermia

**DOI:** 10.3390/ma12172663

**Published:** 2019-08-21

**Authors:** Anastasios Kotoulas, Catherine Dendrinou-Samara, Mavroeidis Angelakeris, Orestis Kalogirou

**Affiliations:** 1 Department of Physics, Aristotle University of Thessaloniki, 54124 Thessaloniki, Greece; 2 Department of Chemistry, Aristotle University of Thessaloniki, 54124 Thessaloniki, Greece

**Keywords:** iron oxide magnetic nanoparticles, polyols, thermal decomposition, solvothermal, magnetic particle hyperthermia

## Abstract

A study of the influence of polyols, with or without an additional reducing agent, on crystallites’ size and magnetic features in Fe_3_O_4_ nanoparticles and on their performance in magnetic particle hyperthermia is presented. Three different samples were synthesized by thermal decomposition of an iron precursor in the presence of NaBH_4_ in a polyol. So far, triethylene glycol (TrEG) and polyethylene glycol (PEG 1000 and PEG 8000) that exhibit different physical and chemical properties have been used in order to investigate the influence of the polyols on the composition and the size of the NPs. Additionally, the presence of a different reducing agent such as hydrazine, has been tested for comparison reasons in case of TrEG. Three more samples were prepared solvothermally by using the same polyols, which led to different crystallite sizes. The magnetic core of the nanoparticles was characterized, while the presence of the surfactant was studied qualitatively and quantitatively. Concerning the magnetic features, all samples present magnetic hysteresis including remanence and coercivity revealing that they are thermally blocked at room temperature. Finally, a study on the influence of the MNPs heating efficiency from their size and the field amplitude was accomplished. In our polyol process the main idea was to control the specific loss power (SLP) values by the nanoparticles’ size and consequently by the polyol itself.

## 1. Introduction

Iron oxide nanoparticles have received much attention the past decades because of their numerous applications in a wide range of scientific fields such as catalysis [1], environment [2], magnetic resonance imaging [3,4], magnetic hyperthermia [5,6] and targeted drug delivery [7,8]. Nanostructured iron oxide can be synthesized by a variety of wet chemistry methods. Generally, since the approach of wet chemistry methods is aggregation of atoms produced in solution into nanoparticles, good control of the reaction, of the size of the particles, and the size distribution may be achieved. The most widely used methods of iron oxide nanostructuring are thermal decomposition [9,10], sometimes combined with the polyol process [11,12], and solvothermal method [13,14,15,16], where polyols may also be used as well. Some of the most common precursors that have been used in iron oxide nanostructuring are iron (II) acetylacetonate [17], iron oleate [18], iron (II) acetate [19], iron nitrate nonahydrate [20], and iron pentacarbonyl [21,22]. Iron pentacarbonyl has been used in the preparation of iron nanoparticles [23] or in the preparation of iron/iron oxide core/shell nanoparticles [24,25]. A similar core/shell structure has been obtained by using iron (II) or iron (III) chloride [26,27,28,29]. In our synthetic protocol, we used both thermal decomposition and solvothermal method with iron (III) acetylacetonate (Fe(acac)_3_), a common Fe precursor [9,10,30] and polyols.

The polyol process is a quite popular synthetic procedure since each polyol may act as a solvent, reducing agent, and surfactant, simultaneously. Generally, this method offers a wide range of reaction temperature values, considering that any long-chain polyol has a different boiling point, which depends on the relative molecular mass, leading to the control of the physicochemical characteristics of the nanoparticles such as crystal phase and size. Magnetite particles in a size range between 82 and 1116 nm have been prepared by Liu et al. [14] by using ethylene glycol (EG). Longer-chain polyols such as triethylene glycol (TrEG) have been used in magnetite nanostructuring [14,15] as well, while even longer polyethylene glycols (PEGs) resulted to same phase nanoproducts [15,16]. Beside magnetite, polyol process has been proved beneficial for other ferrite nanostructures such as cobalt [31] and nickel [32] ferrites. Basti et al. [33] managed to prepare both maghemite and magnetite nanoparticles by diluting iron (II) acetate in diethylene glycol. The main factor in their synthetic protocol which determined the formation of either maghemite or magnetite was the hydrolysis ratio, defined by the nominal water per iron molar ratio. 

The inherent properties of polyol influence the size of the synthesized nanoparticles; Gonçalves et al. [34] have synthesized colloidal magnetite nanocrystals in the range of 5.8–11 nm by using TrEG, PEG-1000, and PEG-8000. Moreover, the long-chain polyols favor the formation of nanostructured microspheres; Zhao et al. [35] have solvothermally prepared in one step hierarchically nanostructured magnetic hollow spheres of magnetite and maghemite in the presence of PEG-2000, while Cao et al. [36] have prepared iron oxide hollow spheres by a similar method. Meanwhile, previously it has been shown by us that these long-chain polyols favor the formation of large cobalt based nanoparticles [31].

The size of the particles is a fundamental factor which determines to a large degree their magnetic behavior and, consequently, the specific loss power (SLP), a figure of merit in magnetic particle hyperthermia. There are two crucial values for the particles’ diameter: The critical diameter (D_cr_) which segregates the nanoparticles to multi domain (D > D_cr_) or single domain (D < D_cr_) particles; and the superparamagnetic critical diameter (D_spm_), which is the limit between the superparamagnetic (D < D_spm_) and the thermally blocked regime (D > D_spm_). In literature, a wide range of both diameter values has been reported [37,38,39,40].

SLP is the quantification of power losses during magnetic hyperthermia measurements. The losses originate by a contribution of three processes; the hysteresis losses, the Brownian and Néel relaxation [41]. The SLP values increase with the increase of field amplitude because of the increment of the offered energy [42]. The magnetic losses are proportional to the square of field amplitude for superparamagnetic nanoparticles (SLP~H^2^), while a stronger field dependence (SLP ~ H^3^) is frequently followed for larger nanoparticles [43]. In general, the smaller the particles, the better the agreement with the H^2^ SLP dependence following the low field regime (linear response theory [44]). As particles grow in size, they magnetically harden, thus a more pronounced yet not systematic SLP dependence on H is shown. In some reports, the field amplitude dependence obeys a power law of an intermediate value of 2.5 (SLP ~ H^2.5^). This value is ascribed to the combinatory contribution of two mechanisms; hysteresis losses and relaxation mechanisms [45].

Herein, the preparation of magnetite nanoparticles by means of thermal decomposition and solvothermal method is attempted with the polyol process incorporated with or without additional reducing agents. Four samples (TD1–TD4) were synthesized via the thermal decomposition method and three solvothermally (SV5–SV7). The triethylene glycol (TrEG) in TD1 and polyethylene glycol with relative molecular masses equal to 1000 g/mol (PEG-1000) in TD2 and 8000 g/mol (PEG-8000) in TD3 have been used. Finally, the fourth sample (TD4) was synthesized in TrEG by using hydrazine as the reducing agent, in order to test the influence of hydrazine on the size of the prepared nanoparticles. The same polyols were used for the solvothermally prepared samples SV5, SV6, and SV7, i.e., TrEG, PEG-1000, and PEG-8000, respectively. These polyols exhibit different boiling points and redox potential and their influence in the characteristics of the synthesized nanoparticles is examined. The size of the particles affects directly the magnetic features and, consequently, the SLP values of the samples. Hence, the kind of polyol used affects the magnetic behavior, the SLP values and the field dependence of SLP. Thus, it is demonstrated that the control of the SLP from the polyol is feasible. It has to be noted that the metal precursor as well as the molar ratio of polyol to metal precursor were kept constant in both chemical approaches in order to investigate the effect of the use of reducing agent on the prepared nanoparticles.

## 2. Materials and Methods

### 2.1. Chemical Reagents

All chemicals were used as received without further purification. Iron (III) acetylacetonate (≥97%, M_r_ = 353.2 g/mol) was purchased from Fluka (Munich, Germany), triethylene glycol (>99%, M_r_ = 150.2 g/mol) and PEG-1000 (≥99%) from Merck (Kenilworth, NJ, USA), PEG-8000 (≥99%) and hydrazine hydrate (64%–65%, M_r_ = 50.1 g/mol) from Sigma-Aldrich (St. Louis, MO, USA).

### 2.2. Materials Preparation

Four different samples (TD1–TD4) were produced via the thermal decomposition method, Table 1. For the synthesis of the first one (TD1), all reagents were inserted into the three neck flasks from the beginning of the procedure, whereas for the synthesis of TD2 and TD3 hot injection of the reagents occurred. The procedure that was accomplished for TD1 synthesis comprised the simultaneous insertion in the flask of 40 mL TrEG (300 mmol), 5 mmol Fe(acac)_3_, and 5 mmol NaBH_4_ in 2 mL deionized water. After the insertion, air was removed by Ar flow for 30 min before heating, with a heating rate of 3 °C/min to a maximum temperature of 260 °C, under vigorous stirring (500 rpm). After 1 h, the black solution was allowed to cool down to room temperature and the sediment obtained by centrifugation (6000 rpm) was washed several times first with a mixture of ethanol-acetone and then with distilled water, to remove the byproducts. In this case TrEG played a double role acting as both solvent and surfactant [12]. The whole procedure occurred under Ar flow in order to remove the gas byproducts. The nanoparticles that were produced, were dispersed and stored in hexane. To study the influence of another reducing agent on the size and the structure of the nanoparticles, TD4 was synthesized similarly to TD1, but instead of NaBH_4_, hydrazine hydrate was used while the molar ratio of the reagents was kept constant.

Sample TD2 was synthesized in a slightly different way. First, 20 g of PEG-1000 were inserted in the three-neck flask and air was removed with Ar flow for 30 min. The polyol was slightly heated, and after its melting, a solution of acetone and 2.5 mmol of Fe(acac)_3_ was injected. An aqueous solution of 2.5 mmol NaBH_4_ (2 mL) was injected with a syringe at 100 °C. The solution was maintained at 130 °C for 1 h to remove acetone and water, while further heated to a maximum temperature of 260 °C with a heating rate of 3 °C/min. After 1 h of refluxing, the black solution was left to cool down to room temperature. The nanoparticles were received and stored as mentioned above.

The TD3 sample was produced with the double hot injection of an acetone (8 mL) solution that contained 2.5 mmol Fe(acac)_3_ and 2.5 mmol NaBH_4_ in deionized water (2 mL). The solutions were injected to 20 g of PEG-8000 at 100 °C. Before reaching 260 °C, with a heating rate of 3 °C, the temperature remained constant at 130 °C for 1 h to remove water and acetone. At the moment of hot injection, the temperature instantly fell to 65 °C.

Samples SV5–7 were prepared solvothermally. The solutions were hermetically closed in an autoclave Teflon vessel and heated at 200 °C for 24 h, with a heating rate of 3 °C/min. Fe(acac)_3_ was used as a precursor, while TrEG, PEG-1000, and PEG-8000 were used as the solvent, reducing agent, and surfactant, in a triple role, for samples SV5, SV6, and SV7, respectively. The molecular proportion between the precursor and the surfactant in pairs TD1-SV5, TD2-SV6, and TD3-SV7 was kept constant, 1.25/75, 1.25/10, and 1.25/1.25, respectively. Table 2 presents the synthetic conditions of the solvothermally produced samples.

### 2.3. Characterization Techniques

The crystalline structure and morphology of the nanoparticles were examined by means of X-ray powder diffraction patterns (XRD) recorded at Bragg–Brentano geometry using a two cycle Rigaku Ultima+ powder X-ray diffractometer (Tokyo, Japan) with a CuKa radiation operating at 40 kV/30 mA. The 2θ angular range was 10°–90°, the step size 0.05°, and the step time 2 s; transmission electron microscopy (TEM) was performed by drop-casting the colloidal dispersions onto the carbon-coated Cu grids after sonication for 1 h in hexane. TEM images were acquired in a JEOL 2011 UHR electron microscope (Tokyo, Japan) with a 0.194 nm resolution, operated at 200 kV.

Infrared (IR) spectra (400–4000 cm^−1^) were recorded on a Nicolet FT-IR 6700 spectrometer (Thermo Scientific, Waltham, MA, USA) with samples prepared as KBr pellets. Thermogravimetric analysis (TGA) measurements of powder particles were carried out (SETA-RAM SetSys-1200, Setaram Instrumentation, Caluire-et-Cuire, France) in the range from room temperature to 800 °C at a heating rate of 10 °C/min under N_2_ atmosphere.

Magnetic measurements were performed using a vibrating sample magnetometer (VSM-1.2 T Oxford Instruments, Abingdon, UK) at room temperature, at a maximum field of 1.1 T. Magnetic particle hyperthermia was carried out at an AC magnetic field at f = 765 kHz, at different concentrations from 0.25 to 2 mg/mL, and field amplitudes of 15, 20, 25, and 30 mT. Each colloidal was placed in the center of a water-cooled induction coil connected to an AC field generator (SPG-10: Ultrahigh Frequency Induction Heating Machine, Shuangping Corp., Shenzhen, China), using a GaAs-based fiber optic probe.

## 3. Results

### 3.1. Structural Characterization

#### 3.1.1. Transmission Electron Microscopy

**Figure 1:**
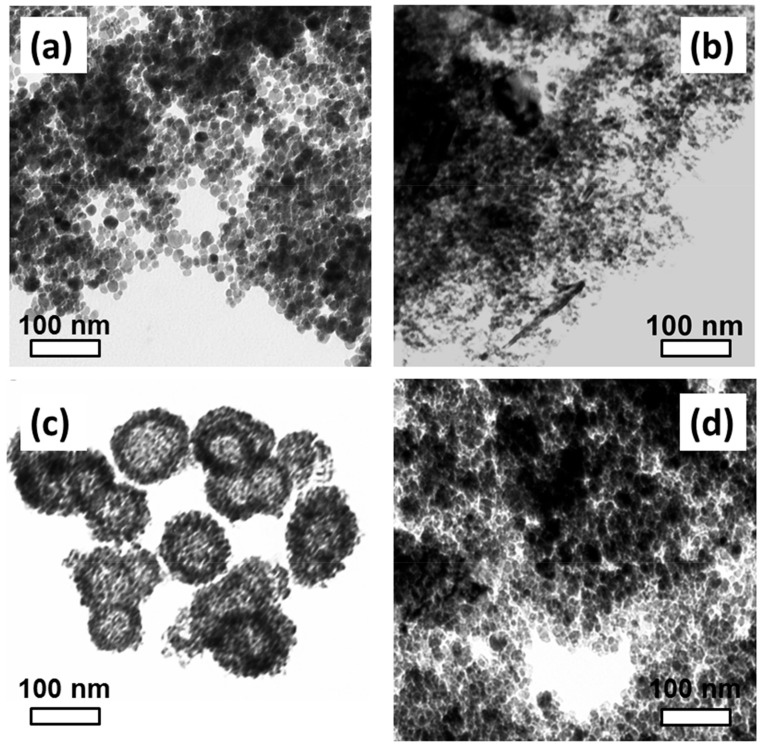
Transmission electron microscopy (TEM) images of samples (**a**) TD1, (**b**) TD2, (**c**) TD3, and (**d**) TD4. The TD3 nanoparticles, which were synthesized with PEG-8000 as surfactant, are self-organized in microspheres.

Transmission electron microscopy (TEM) was performed for the four samples synthesized by thermal decomposition (TD1–TD4), Figure 1. TD1 and TD4 nanoparticles, namely the ones that were prepared with TrEG, tend to be less aggregated than the nanoparticles of TD2 and TD3. Specifically, in TD3, the nanoparticles seem to be organized in spherical structures (Figure 1c). Generally, self-assembled structures tend to be formed by using PEG with relatively high molecular weight like PEG-8000 [46].

A similar morphology as that of Figure 1c has been reported previously. Zhao et al. [35] prepared solvothermally magnetite nanoparticles, which were self-organized in 300 nm sized microspheres. In their chemical procedure, ethylene glycol as both reducing agent and solvent, FeCl_3_ as precursor, PEG-2000 and a small quantity of polyethyleneimine, have been used. PEG-2000 seems to favor the formation of these hierarchically structured microspheres. Hollow hierarchical α-Ni(OH)_2_ superstructures have been prepared by Song et al. [47] solvothermally by using PEG as surfactant. By heating these superstructures in air, they were transformed into nickel (II) oxide nanoparticles. PEG was also used for hollow spheres synthesized by iron oxide (Fe_3_O_4_ or/and γ-Fe_2_O_3_) nanosheets [36].

The long-chain polyols favor the formation of cobalt nanostructured microspheres, as well. Qiao et al. [48] prepared solvothermally hierarchical structured hollowed spheres by using the copolymer PEG-PPG-PEG as surfactant and ethylene glycol as solvent. PPG is poly-propylene glycol, a high relative molecular mass copolymer of 2800 g/mol. We follow a similar procedure, in the sense that it was accomplished in one step by using the polyol method. It has been confirmed that the cobalt nanoparticles which have been initially synthesized from the simultaneous polyol effect as solvent and reducing agent, have been gradually aggregated. The prepared nanoparticles are partly stabilized and, if the reaction time is long enough, the formation of hollow microspheres is favored by localized Ostwald ripening. When the reaction time is 3 h or less, only nanoparticles are produced, which probably cannot further be organized in microstructures. Conversely, when the reaction time elongates to 12 h the hollow spheres are formed. From the previous discussion and our results (see Figure 1), it seems that the polyethylene glycols with high molecular mass favor the formation of hierarchical superstructures. It seems that the long chain structures, such as the PEG-PPG-PEG copolymer can be adsorbed on the surface of more than one nanoparticle, as it has been demonstrated by Qiao et al. [48], and self-organize to form microspheres.

**Figure 2:**
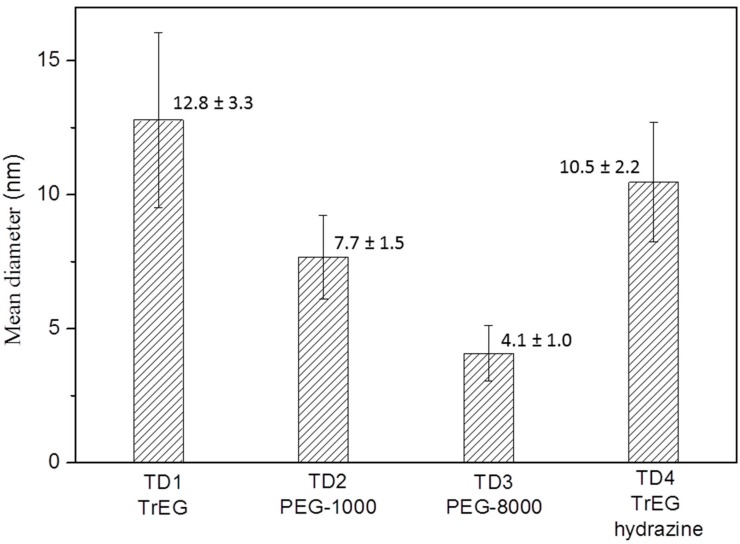
The mean diameter and its standard deviation of nanoparticles in samples TD1–TD4.

Figure 2 shows the mean size values of the TD1–TD4 nanoparticles, as measured from ΤΕΜ images. Concerning sample TD3, only nanoparticles which are clearly segregated were taken into account, namely only the nanoparticles that are located within the spheres. It seems that as the value of relative molar mass of the surfactant increases, the mean size of the nanoparticles decreases, while the size dispersion is not significantly affected. 

Given that the reducing ability of a polyol depends on its molecular weight, among triethyleneglycol, PEG-1000, and PEG-8000, triethyleneglycol shows the highest reductive ability and PEG-8000 the weakest [46,49]. When an additional reducing agent participates in the synthesis, the mechanism which affects the system iron precursor-NaBH_4_-polyol is quite complicated. Generally, it is accepted that NaBH_4_ is a more efficient reducing agent than a polyol [50]. Consequently, the use of NaBH_4_ affects drastically the mean size of the particles by increasing the decomposing rate of the precursor and therefore, the nucleation rate of the nanoparticles; the increase of nucleation rate leads to a decrease of the size of the nanoparticles [51,52]. Glavee et al. [53] proposed a chemical pathway which describes the formation of iron nanocomposites by the reduction of iron (II) or iron (III) bromide in aqueous or non-aqueous media. Concerning the reduction of iron (III) bromide in non-aqueous medium, their idea relies on the formation of an intermediate complex, (L)_n_Fe(BH_4_)_3_, where L represents diethylene glycol dimethyl ether (diglyme). The final product was FeB nanocrystals, which in the presence of oxygen and heating gave α-Fe nanoproducts. In our experiments, the pathway could give the Fe atoms, which were oxidized in magnetite and were assembled to magnetite nanoparticles. On the other hand, under high temperatures polyols oxidized to various aldehydes and ketones species [54] that can be further reduced by NaBH_4_ to their respective alcohols [55]. Therefore, it is difficult to find out which path is dominant. Certainly, the addition of an extra reducing agent, such as NaBH_4_, accelerates the reduction of Fe(acac)_3_ and Fe nuclei increase leading to smaller nanoparticles.

Considering that in all samples (TD1–TD3) the same amount of NaBH_4_ has been used, the size variation (4.1 to 12.8 nm) was attributed to the polyols TrEG, PEG-1000 and PEG-8000, respectively. The mean size values as measured from TEM images and the corresponding standard deviation values are given in Figure 2. Additionally, when NaBH_4_ is replaced with hydrazine (TD4) the size of the nanoparticles were found to be similar to TD1, which indicated further impact of the extra reducing agent. 

#### 3.1.2. XRD Measurements

**Figure 3:**
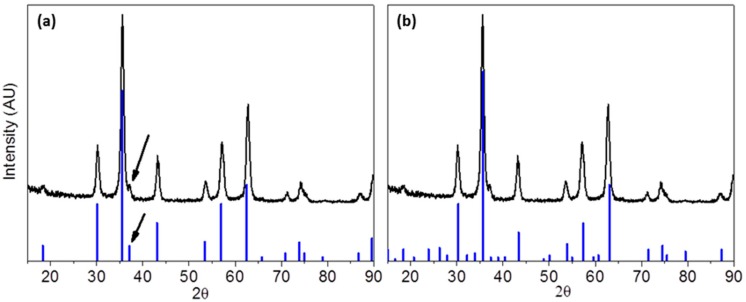
X-ray diffraction (XRD) pattern of sample TD1 in comparison with the peaks of two iron oxides: (**a**) magnetite (PDF #19-0629) and (**b**) maghemite (PDF #25-1402). The arrow shows the presence of a peak that corresponds to magnetite.

The crystalline structures of the metallic core of all samples were determined by XRD. Figure 3 shows the XRD pattern of sample TD1. Generally, the phase characterization of an iron oxide only by XRD is quite intractable because of the identical patterns of the most common iron oxides; magnetite (Fe_3_O_4_) and maghemite (γ-Fe_2_O_3_). However, concerning samples TD1–TD4, it seems that magnetite is the dominant phase, although the presence of a small amount of maghemite cannot be excluded, since it is well known that magnetite slowly oxidizes to maghemite. This conclusion arose from the presence of a weak peak at 37.1°, which is noted with an arrow in Figure 3.

Magnetite at 37.1° shows a peak with relative intensity 8%, while maghemite at the same angle shows a four-time weaker peak (2%) and probably if TD1 consisted of maghemite this peak would be unrevealed. Additionally, if a 2% peak could be detected, then the two next distinct peaks of maghemite at 38.8° and 40.4° should be detected as well. The peak that appears at 18.6° could correspond to either magnetite or maghemite. However, maghemite should be excluded since in that case two more peaks at 23.8° and 40.4°, with the same relative intensity with the one at 18.6°, that correspond to maghemite, should be detected. A considerable number of researchers concluded in similar results by using PEGs in the preparation of iron oxide nanoparticles. Wan et al. [12] have synthesized iron oxide nanoparticles by thermally decomposing Fe(acac)_3_ in TrEG at 278 °C under inert atmosphere without using any extra reducing agent. The XRD patterns have revealed the exclusive presence of magnetite, a result that has been confirmed by HRTEM. Maity et al. [11] repeated the production of iron oxide nanoparticles by the same method, i.e., thermal decomposition of Fe(acac)_3_ in TrEG at 280 °C, and Gonçalves et al. [34] synthesized magnetite nanoparticles in high molecular weight polyols.

**Figure 4:**
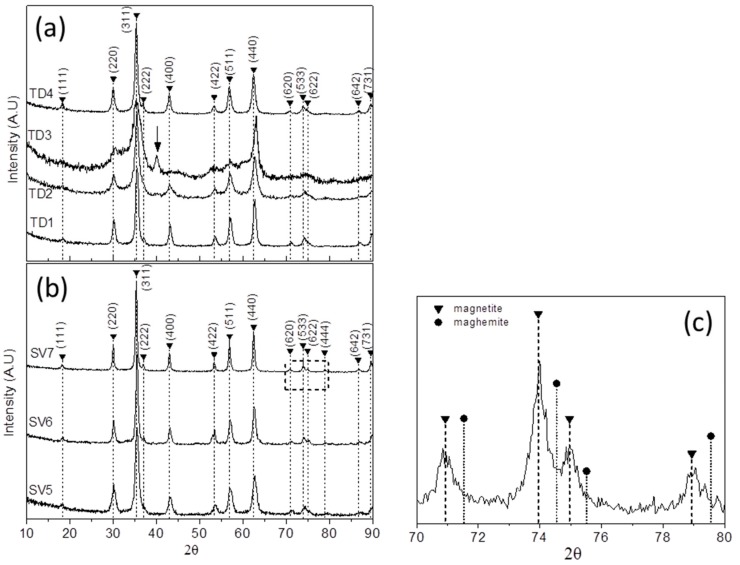
The XRD patterns of (**a**) the thermal decomposition samples TD1, TD2, TD3, TD4 and (**b**) the solvothermal samples SV5, SV6, and SV7. The dominant phase in all samples is magnetite. (**c**) An enlarged area of the Figure 4b from 70° to 80° certifies that the solvothermally synthesized samples consist almost exclusively of magnetite.

Figure 4a shows the XRD patterns of the samples TD1–TD4 for comparison. It seems that all of them consist of the same crystal phase (magnetite) and the peaks correspond to the crystal planes (111), (220), (311), (222), (400), (422), (511), (440), (620), (533), (622), (642), and (731). Specifically, the peak at 37.1° that is assigned as (222) crystal plane is clearly visible in the patterns of TD1 and TD4, on account of the weaker background noise. On the contrary, in the patterns of TD2 and TD3 that peak is barely visible.

The XRD pattern of TD3 is quite unusual. A peak at 40.3°, which is neither related to magnetite nor maghemite, is present. Moreover, the weaker peaks are not revealed, as a result of the enhanced background noise, and the peaks are broader than those of the other patterns. The aforementioned peak, which is accentuated with an arrow in Figure 4a, might correspond to iron (III) oxide hydroxide, FeO(OH), an intermediate compound of the chemical reaction where magnetite is formed by Fe(acac)_3_. The XRD pattern of FeO(OH) nanoparticles [56] show a strong peak at 40.5°. The formation of magnetite NPs by Fe(acac)_3_, at room temperature and 78 °C in the presence of aqueous solution of NaBH_4_ has been suggested by Yathindranath et al. [57]. Based on that, a trivalent iron cation of Fe(acac)_3_ coalesces with three hydroxyl anions to form Fe(OH)_3_ as follows:

Fe^3+^ + 3 OH**^−^** → Fe(OH)_3_

The hydroxyl ions presence is ensured by the aqueous conditions and the presence of NaBH_4_. In the case of TD3, synthesis was performed by hot injection at 100 °C of the acetone dissolved precursor (Fe(acac)_3_) and the injection of NaBH_4_ diluted in water. The hot injections of the reactants with the simultaneous fall of temperature down to 65 °C, simulated the appropriate chemical conditions that were described above. Specifically, a higher pH value was achieved, namely by the presence of hydroxyl cations. Subsequently, Fe(OH)_3_ decomposes to FeO(OH) and water as follows:

Fe(OH)_3_ → FeO(OH) + H_2_O

Simultaneously, part of the trivalent iron cations is reduced to divalent cations because of the presence of NaBH_4_. The divalent iron cations and the hydroxyl anions react to form iron (II) hydroxide:

Fe^2+^+2 OH**^−^** → Fe(OH)_2_

Finally, the two hydroxides react to form magnetite:

2 FeO(OH) + Fe(OH)_2_ → Fe_3_O_4_ + 2 H_2_O

The FeO(OH) formation occurs at about 80 °C and the presence in the final product of TD3, is related to its stability and the followed procedure for this sample. However, no presence of FeO(OH) was detected in the XRD patterns of samples TD1, TD2, and TD4, indicating that even small changes at the synthetic procedure are crucial. In so, for TD1 and TD4 all reagents were inserted in the flask before heating while for TD2 the injection of the aqueous solution of NaBH_4_ was at about 100 °C and raised quickly to 130 °C. Thus we assume that all FeO(OH) quantity that might be produced at low temperature reacted with Fe(OH)_2_ to form magnetite at higher temperature. Nerveless even if a very low amount of FeO(OH) remained unreacted, it could not be detected by the XRD technique. 

In Figure 4b XRD patterns of samples SV5, SV6, and SV7 are presented. In Figure 4c a magnification of the XRD pattern of SV7 is presented which includes only the reflections between 70° and 80°, and the magnetite and maghemite peaks are noted. The solvothermally prepared samples may consist of magnetite exclusively. For argument’s sake, the SV6 and SV7 XRD patterns show a clear peak at 37.1°, a precise evidence of magnetite as mentioned above. Besides, in the enlarged area (Figure 4c) the maghemite reflections are too far in contrast to the magnetite ones. By comparing Figure 4a,b an extra peak appears at 78.9° in the patterns of Figure 4b, which correlates with the reflection of (444) crystal plane of Fe_3_O_4_ phase. This is a very weak peak, with a relative intensity of only 2%, which is not illustrated in the XRD patters of TD1–TD4 (Figure 4a) as a result of the background noise. The other peaks of the patterns correlate with the (111), (220), (311), (222), (400), (422), (511), (440), (620), (533), (622), (642), and (731) crystal planes of magnetite.

The solvothermal synthesis of magnetite nanoparticles with no presence of maghemite by applying the polyol process has been already confirmed, elsewhere; Yan et al. [16] prepared Fe_3_O_4_ nanoparticles by using PEG-6000 and sodium dodecyl sulfate (SDS). FeCl_3_·6H_2_O was used as a precursor and the reaction temperature was 180 °C for 6–72 h. Though the reaction was accomplished under atmospheric oxygen, the maghemite formation was deterred due to the appropriate reductive conditions. In samples SV5, SV6, and SV7, correspondingly, thanks to the reductive conditions that were imposed by the polyols, the maghemite formation was prevented despite the presence of atmospheric oxygen inside the reaction vessel. From our results, it seems that both methods, which are combined with the polyol process, favor the formation of magnetite nanoparticles. In thermal decomposition the polyols and NaBH_4_ ensured the appropriate reductive conditions, while in solvothermal, only the presence of polyols ensured the same results.

The samples that were solvothermally prepared illustrate the size dependence from the polyol molecular weight; an increase of the relative molecular mass led to bigger mean size crystallites in accordance to Gonçalves et al. [34] results. It is worth noting that in solvothermal method NaBH_4_ was not used; therefore, the role of reducing agent was played only by polyol itself. Table 3 shows the crystallites’ sizes of all samples, as calculated by Scherrer equation, which was applied for the most intense peaks of the XRD patterns (Figure 4):

(1) $$D=\frac{0.9\cdot \lambda }{b\cdot cos\theta },$$

where *D* is the mean crystallites’ size, *λ* is the radiation wavelength, *b* is the full width half maximum, and θ is the half angle of each peak. The estimation of *b* was done by Gaussian fitting of the peak with the highest intensity. Since in the TD samples the nanoparticles’ mean size values, which were calculated by the TEM images, concur with the crystallites’ mean size values, the inferred conclusion is that the nanoparticles probably consist of single crystals.

Comparing the sizes of the crystallites between samples that were synthesized with the same polyol but with different method (TD1-SV5, TD2-SV6, and TD3-SV7), a differentiation is observed. Unless the exception of TD1-SV5, where the solvothermally structure crystallites are smaller, in the other pairs they are quite larger. The increased reaction duration from 1 h in thermal decomposition method to 24 h in solvothermal could be the reason for this size increase, since the crystallites grow for larger period. A second reason could be the more reductive environment of TD samples because of NaBH_4_, the presence of which led to the creation of larger numbers of nuclei and consequently to smaller crystallites. 

#### 3.1.3. Infra-Red Spectroscopy

**Figure 5:**
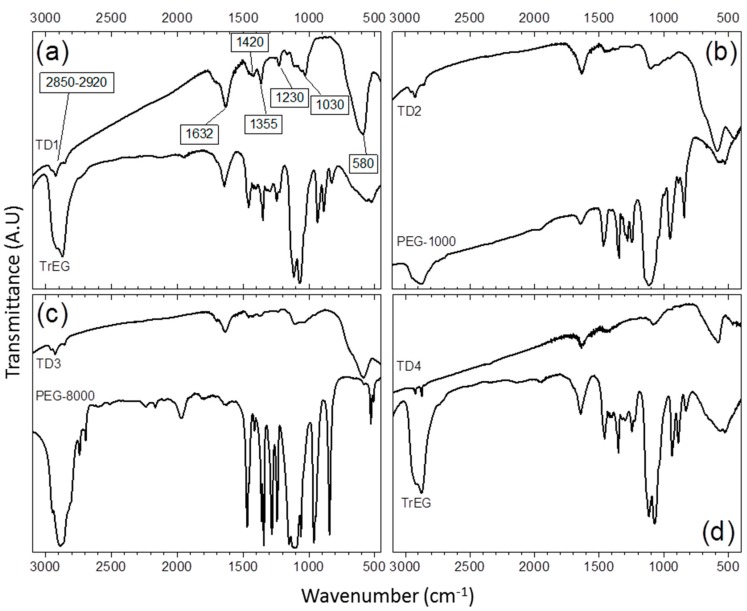
The transmittance IR spectrum of thermal decomposition samples. (**a**) Sample TD1. The peaks that are assigned to specific stretching or bending vibrations of the bonds of TrEG molecule are noted. (**b**) IR spectrum of TD2, (**c**) TD3, and (**d**) TD4.

The existence of the organic coating on the surface of the NPs has been confirmed by the FTIR spectra of the samples (Figure 5). The common nature of the polyols as surfactants is indicative in all cases. All peaks appear slightly shifted in comparison with the pure FTIR spectra of the polyols, indicating the binding of the surfactants on the surface of the NPs. The peaks at 2850–2920 cm^−1^ and at 1420 cm^−1^, demonstrate the presence of methyl groups and are assigned to stretching, *ν*(C–H), and bending, *δ*(C–H), vibrations, respectively. The peak at 1632 cm^−1^ is associated to hydrogen bonding between the neighboring surfactants and/or the glycolate derivatives confirming the presence of oxidized species of the polyols on the surface of the NPs [46]. The peak at 1355 cm^−1^ is assigned to bending vibrations of carbon and hydrogen atoms of CH_3_. Finally, the multiple peaks at about 1030 cm^−1^ correspond to C–O–C bending vibrations and the peak at 1230 cm^−1^ is assigned to C–O–C twisting mode. Conversely, the peak at 580 cm^−1^ is assigned to stretching vibrations of tetrahedral sites of iron atoms and oxygen of magnetite [11,57].

#### 3.1.4. Thermogravimetric Analysis

**Figure 6:**
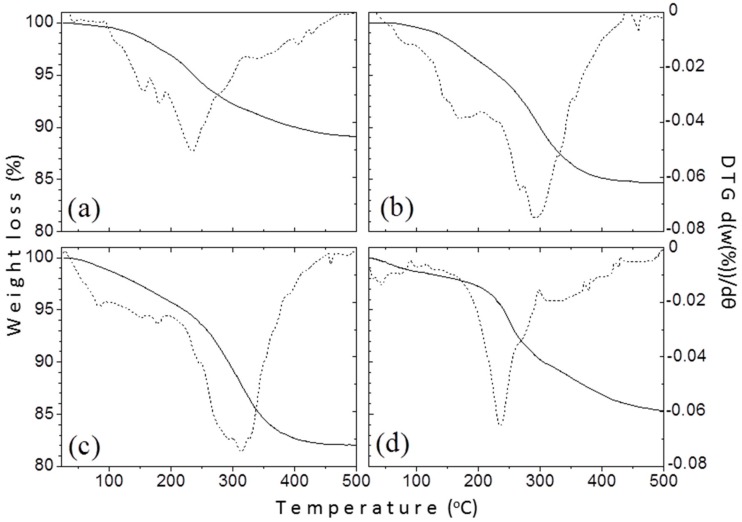
Thermogravimetric analysis (TGA) measurements for the samples: (**a**) TD1, (**b**) TD2, (**c**) TD3, and (**d**) TD4. The continuous line represents the % mass loss versus temperature, while the noncontinuous one the derivative of the mass loss versus temperature.

Thermogravimetric analysis (TGA) was conducted to calculate the amount of the organic coating on the surface of the produced NPs. Figure 6 shows the TGA curves of samples TD1–TD4. The continuous line represents the relative mass (in comparison with the initial mass) of each sample versus temperature, while the dust one represents the derivative of relative mass versus temperature. The heating procedure occurred under inert atmosphere (N_2_) to avoid oxidation of the nanoparticles and the consequent increase of their masses. The horizontal axis of the graphs was drawn up to 500 °C, because at higher temperature values the mass of each sample was almost constant. In any case, the curves seem quite even, therefore the mass loss occurred gradually. The values of the mass loss were found similar at 11, 15.4, 18.3, and 14.9 wt.% for TD1, TD2, TD3, and TD4, respectively. For all samples, the decomposition of the surfactants begins at around 200 °C and finishes right after 400 °C. For samples TD2 and TD3 synthesized in PEG, two weight losses can be observed, indicating a bi-layer formation of the organic coating as also confirmed elsewhere [58]. This is a common phenomenon for high molecular weight surfactants like PEG-1000 and PEG-8000 that curl around the surface of the nanoparticle.

### 3.2. Magnetic Characterization and Hyperthermia

#### 3.2.1. VSM Measurements

**Figure 7:**
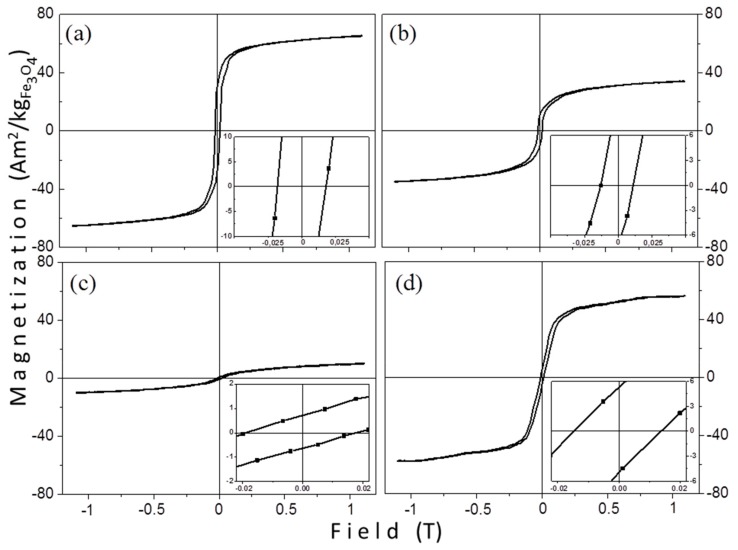
Room temperature hysteresis loops of samples: (**a**) TD1, (**b**) TD2, (**c**) TD3, and (**d**) TD4.

Room temperature magnetization measurements were performed on powder samples. Figure 7 shows the hysteresis loops of the samples that were prepared by the thermal decomposition method, revealing that the MNPs are thermally blocked. However, their macroscopic magnetic parameters are quite different. In Figure 7 the vertical axis in all graphs is in the same scale, to facilitate a direct comparison of the hysteresis loops. The samples do not saturate at the maximum applied field (1.1 T) and a high field susceptibility above 0.5 T is observed. Table 4 contains all magnetic features of the samples. Mass magnetization values are given with respect to the iron-oxide mass (Fe_3_O_4_). The saturation magnetization values were determined from the magnetic moment of the sample at the largest applied field (1.1 T). We assume that the unexpected large coercivity values for such small particles originate from dipolar interactions and/or from the existence of some larger nanoparticles which are classified to multi-domain particles. Both reasons are thoroughly discussed below.

The saturation magnetization values are 73.7, 66.0, 40.2, and 12.6 Am^2^/kg_Fe3O4_ for TD1, TD4, TD2, and TD3, respectively. These scaled values were obtained by calculating the actual Fe_3_O_4_ mass after extracting the surfactants’ mass as obtained from the TG measurements and this gives 80, 72, 44, and 14% of the saturation magnetization of bulk magnetite (92 Am^2^/kg [59]). In Table 4 a clear tendency is observed; the decrease of the mean size of the nanoparticles led to the decrease of saturation magnetization. This could be attributed to surface effects, which are more effective in the smaller nanoparticles [60]. A significant decrease of saturation magnetization with the decrease of mean size is observed. In maghemite nanoparticles a similar trend has been proved [61]. For these particles with sizes ranging from 12 to 3.5 nm, M_s_ values from 69.6 to 2.8 Am^2^/kg have been reported. The tiny M_s_ values are attributed not only to surface effects, but also to the existence of internal canted spins [61].

The surface effects are related to the percentage increase of the surface spins of the nanoparticles, when the surface-to-volume ratio increases. Magnetite nanoparticles with small sizes, 3, 5, and 7 nm, have been prepared but the reported room temperature saturation magnetization values, normalized considering the Fe content, are high: 135, 152, and 173 Am^2^/kg_Fe_, respectively [62]. Concerning samples TD1–TD4 a relation between saturation magnetization and the mean size of the nanoparticles exists. Specifically, for TD3, where the M_s_ value reduces sharply, it seems that probably there is an internal spin canting contributing as well in the reduction of magnetization. Contribution in the M_s_ sharp decrease could be also attributed to the existence of the non-magnetic phase FeO(OH).

On the other hand, the saturation magnetization is not always affected significantly by the size of nanoparticles. Guardia et al. [63] have prepared magnetite nanoparticles with sizes 6, 10, and 17 nm by using oleic acid as the surfactant, where the values of saturation magnetization were 79, 81.8, and 84 Am^2^/kg, respectively. These values are slightly affected by the size of nanoparticles. The main factor responsible for this irregularity is the presence of oleic acid, where a decrease in the surface spins disorder is generated by its molecules. Guardia et al. suggested that the source of the decrease of the disorder lies in the effect of the crystal field. This field, which interacts with the oxygen anions of oleic acid molecules, affects the surface iron cations. Thereby, the surface spins arrange in accordance with the internal ones. This surface spin rearrangement leads to a disorder decrease and, subsequently, to a size dependency of the saturation magnetization.

Table 4 shows the coercivity of nanoparticles with values 17.8, 14, 12.4, and 18.4 mT for samples TD1, TD4, TD2, and TD3, respectively. In the case of TD1, TD2, and TD4, where the sizes of the nanoparticles were 12.8, 10.5, and 7.7 nm, respectively, a rule, in accordance with which a diameter decrease is accompanied by a coercivity decrease, is followed. This only occurs for diameters below a critical value (D_cr_) or, in other words, in single domain nanoparticles. Generally, coercivity increases when the diameter of a particle decreases until a critical diameter, where the coercivity reaches a maximum. The critical diameter is the limit between single domain and multi domain particles. For decreasing diameters of single domain particles, the coercivity decreases and reaches zero when the nanoparticle enters the superparamagnetic regime. The limit between superparamagnetism and ferromagnetism is called superparamagnetic critical diameter (D_spm_).

The values of critical diameter of magnetite nanoparticles differ significantly in literature. Krishnan et al. [37] report a value of 83 nm by theoretical calculations based on a previous publication of Kittel et al. [64], which concerned spherical and monocrystal nanoparticles. An even larger value, 128 nm, has been estimated by Lu et al. [38]. According to theoretical calculations, D_cr_ is expressed by the equation:

(2) $$D_{cr}\approx 18\frac{\sqrt{AK_{eff}}}{\mu_{0}{Μ}_{s}^{2}}$$

where *A* is the exchange constant, *K*_eff_ is the effective anisotropy constant, μ_0_ is the permeability of vacuum, and *M_s_* is the saturation magnetization. Stephen et al. [39] reported values between 70 and 150 nm. Conversely, Verges et al. [40] reported two values significantly lower in comparison with the previous ones. The first one was found 35.4 nm and has been calculated by an equation originally proposed by Kittel [65]. The second one was found 29.7 nm and was calculated by an equation that was proposed by Brown [66].

In any case, by considering whichever value of the above as the critical diameter, nanoparticles TD1, TD2, and TD3 are much lower in diameter (7.7 to 12.8 nm), so they may be safely classified as residing below the limit of single domain particles and most probably below the superparamagnetic/thermally blocked size. Thus, they should not show any hysteresis and, consequently, no remanence and coercivity should be observed. It was assumed that either in the size ensemble there are also larger particles which are above the superparamagnetic size limit and give the hysteresis or that there are dipolar interactions between the particles resulting in bigger aggregates. TD3, for which while the diameter takes the lowest value (4.1 nm), the coercivity takes the highest one (18.4 mT). The origin of this irregularity may be due to the structuring of larger extent clustered spheres from the nanoparticles. The enhanced coercivity in magnetite microspheres in comparison with the nanoparticles from which they consist, has been observed by Zhang et al. [67] as well. Specifically, they have reported 43.9 mT coercivity for microspheres, while the corresponding value for the nanoparticles was 17.8 mT; an outcome that was attributed to their novel morphology. Not only in magnetite microspheres the coercivity enhancement has been noted. Zhu et al. [68] have synthesized cobalt hierarchical nanostructured microspheres and found an enhanced coercivity; they attributed the cause of this enhancement to their morphology. This unexpected result may be due to dipolar interactions between the nanoparticles. Generally, the interactions depend on the distance between the particles. Particularly, when the distance decreases, the interactions increase, as it has been found in ferrofluids systems [69]. An increase in nanoparticles’ concentration would lead to an increase of coercivity, due to enhancement of dipolar interactions between the nanoparticles.

Nadeem et al. [70] have prepared maghemite nanoparticles with a mean size of 4 nm; two samples of as-prepared powder and a compacted one. The compacted sample revealed an enhanced coercivity at 4.2 K in comparison with the coercivity of powder samples (300.8 and 194 mT, respectively). This characteristic has been attributed both to increased dipolar, and exchange interactions. The exchange interactions are significant only for touching nanoparticles across their interfaces. The interactions may increase in the compacted sample because of the decrease of nanoparticles’ separation. In a computational model that has been introduced by Verdes et al. [71] an increase of coercivity with increasing interparticle interactions has been also shown. Duong et al. [72] have synthesized superparamagnetic magnetite nanoparticles with mean size 10.5 nm and filled nanoholes with them. These Fe_3_O_4_-filled nanohole arrays revealed an enhanced coercivity at 300 K, as a result of the dipole interactions between the nanoparticles. These interactions could be dipolar or exchange ones for the touched nanoparticles. The enhancement of coercivity could be explained by Dormann–Bessais–Fiorani model [73,74], where an extra energy factor is introduced in the anisotropy energy barrier (E_A_), as follows:

(3) $$E_{A}=KVsin^{2}\theta +{Β}_{i},$$

where *K* is the anisotropy constant, *V* the particles’ volume, *θ* the angle between the easy axis of the magnetic particle and the direction of the field, and *B_i_* is an energy factor attributed to interparticle interactions. For single domain particles with only dipolar interactions the energy corresponding to dipole interactions is expressed as follows:

(4) $$E_{dip}=-\frac{\mu_{0}m_{0}^{2}}{4\pi d^{3}},$$

where *μ_0_* is the vacuum permeability constant, *m_0_* the magnetic moment of a particle, and *d* the interparticle separation [74]. A decrease in interparticle separation would cause an increase of *E_dip_* and, therefore, a decrease of the anisotropy energy barrier, leading to an enhanced coercivity. Thus, in sample TD3, which consists of nanostructured microspheres, the separation between the particles is smaller in comparison with particles’ separation in TD1, TD2, or TD4 and as a result, the particles’ interactions are enhanced, which probably leads to an increased coercivity.

The value of superparamagnetic diameter differs significantly in literature, as well. A theoretically estimated value could be derived by using the equations [75]:

(5) $$V_{spm}\approx \frac{25k_{B}T}{K}$$

and

(6) $$D_{spm}={\left(\frac{6}{\pi }V_{spm}\right)}^{1/3}$$

In Equations (5) and (6) *k_B_* is the Boltzmann constant, *K* is the anisotropy constant, and *T* the temperature. The *D_spm_* value could receive a wide range of values, since it appears a strong dependence from the anisotropy constant. For example, Yamaura et al. [76] by using these equations found a value of 26 nm, while a typical value derived from magnetic susceptibility measurements is 17 nm [77]. Nevertheless, since the samples TD1, TD2, and TD4 reveal coercivity, besides superparamagnetism, they possess magnetic hysteresis features; especially, concerning TD3 NPs, although the mean diameter takes the lowest value, they show some coercivity, probably because of enhanced dipolar interactions between the nanoparticles, as mentioned above.

**Figure 8:**
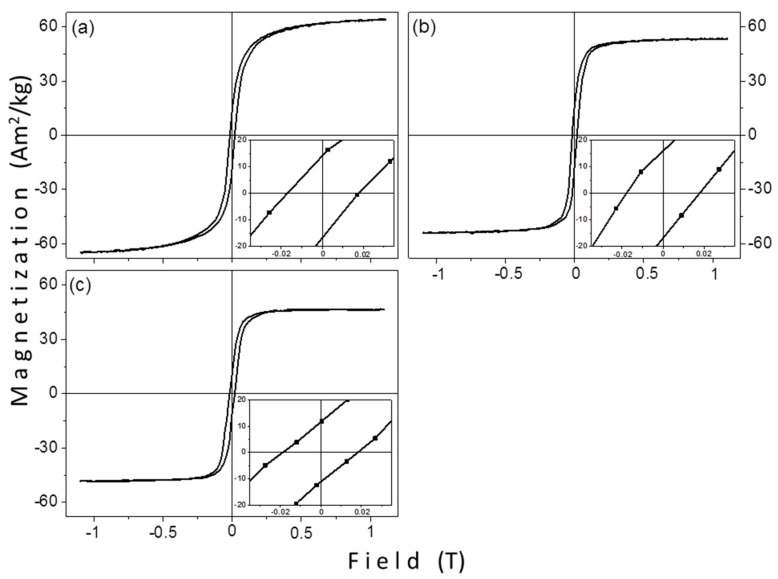
The hysteresis loops of the solvothermally synthesized samples: (**a**) SV5 prepared with TrEG, (**b**) SV6 with PEG-1000, and (**c**) SV7 with PEG-8000.

Figure 8 shows the magnetic measurements of the solvothermally prepared samples, SV5, SV6, and SV7. All of them are thermally blocked at room temperature, like the samples that were prepared by the thermal decomposition method. The values of saturation magnetization are 64.1, 53.3, and 46.3 Am^2^/kg and the coercivity values 17.6, 17.8, and 18.5 mT, for SV5, SV6, and SV7, respectively. The magnetic features are presented in Table 5.

From Table 5 a gradual decrease of saturation magnetization with increasing crystallite size is observed. Thus, for the SV samples the M_s_ vs. size dependence is in the other way, a smaller crystallite size gives a higher M_s_. This decrease could be related to the proportion of the surfactant on the surface of the particles and the increase of the relative molecular mass of the surfactant. A similar behavior was observed in TD1 (TrEG), TD2 (PEG-1000), and TD3 (PEG-8000), where the relative mass loss was 11%, 15.4%, and 18.3%, respectively. Conversely, coercivity is not significantly affected by the surfactant. Its values are almost equal for all samples. The ratio M_r_/M_s_ is not importantly affected from the surfactant, as well. Its values, 25.3%, 30.2%, and 23.8% for SV5, SV6, and SV7, respectively, do not follow any pattern that could be related with the relative molecular mass or the reductive ability of each polyol, since M_s_ and M_r_ values were determined from the maximum applied field (1.1 T) and not from the actual saturation field. In Table 6 the saturation magnetization values of SV5, SV6, and SV7 with the corresponding values of TD1, TD2, and TD3 are compared. The molecular ratio between the precursor and the surfactant remained constant in samples’ pairs. Particularly, in the case of TD1-SV5 pair the molar ratios were 5:300 and 1.25:75, respectively; for TD2-SV6 2,5:20 and 1.25:10 respectively, and for TD3-SV7 2.5:2.5 and 1.25:1.25, respectively.

In both methods a decrease of saturation magnetization when the relative molecular mass of the polyol increases is observed; for TD1–TD3 this decrease is sharper than the decrease of SV5–SV7. For samples TD1, TD2, and TD3, besides the effect of the polyol mass, the size of the particles significantly influenced the saturation magnetization; the size decrease led to an increase of surface spins, hence to a decrease of magnetization. The magnetization decrease in SV5–SV7 samples is observed with increasing the size of the crystallites, an observation which seems discrepant at first glance. Thus, it should be assumed either that the sizes of the particles follow a diverse path (namely their sizes decrease with the increase of the relative molecular mass of the polyol) or that the sizes are insignificantly affected and only the increase of the mass of the adsorbed surfactant affects the saturation magnetization. However, in both cases, the conclusion that can be drawn is that the SV5, SV6, and SV7 nanoparticles have a polycrystalline structure. If the particles were single crystals, then the values of coercivity (17.6, 17.8, 18.5 mT) should notably differ considering that the size of crystallites of the three samples vary considerably (8.6, 13.7, 18.7 nm).

The polycrystalline structure of solvothermally prepared nanoparticles has been already reported. To synthesize PEGylated polycrystalline magnetite nanoparticles, Yuan et al. [15] have followed a similar process, which included more reagents. FeCl_3_·6H_2_O was diluted in a mixture of ethylene glycol and ethanolamine. In that mixture, sodium acetate anhydrate (NaAc) and PEG-2000 were added and the final solution was placed in an autoclave vessel. The reaction took place at 200 °C for 10 h. The synthetic procedure of SV5, SV6, and SV7 comprised only the precursor and different for each sample polyol. Liu et al. [14] have prepared polycrystallic magnetite nanoparticles, as well. They used polyacrylic acid as surfactant and FeCl_3_·6H_2_O as precursor and they synthesized nanoparticles between 82 and over 1000 nm. Generally, the solvothermal synthesis seems to favor the production of nanoparticles with polycrystalline structure under the appropriate reaction conditions.

#### 3.2.2. Magnetic Particle Hyperthermia

**Figure 9:**
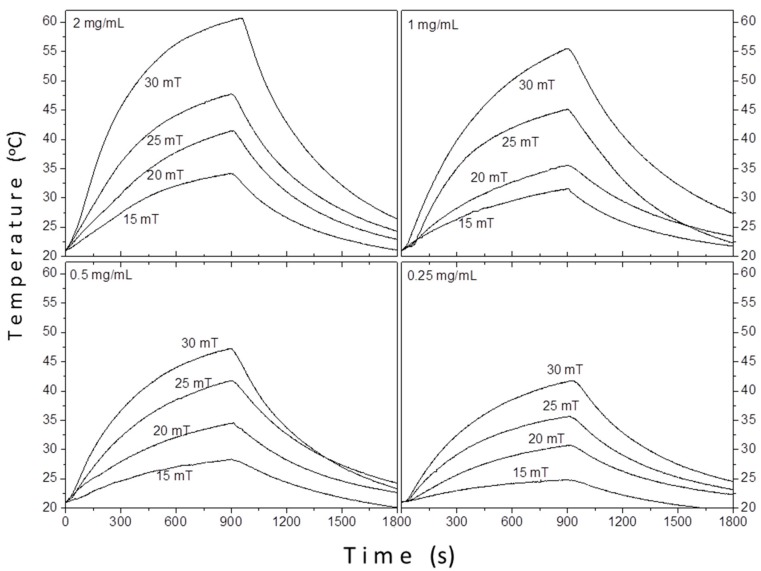
The temperature versus time curves of sample TD1. The temperature increases as long as the magnetic field is applied (900 s).

Figure 9 shows the hyperthermia curves of sample TD1 at four different concentrations (0.25, 0.5, 1, and 2 mg/mL), under four different amplitudes of the applied AC field (15, 20, 25, and 30 mT) and a frequency of 765 kHz.

Apparently, as one can see in Figure 9, the ΔΤ increases either with the increase of the applied field or the increase of the concentration. The contribution of solvent (distilled water) in the raise of temperature was deducted in each case; the values refer exclusively to the contribution of the nanoparticles. 

Besides the concentration and the field amplitude, the rise in the temperature is affected by the conditions under which the measurements are carried out. During the experimental sequence, the temperature was recorded every 0.4 s by using a GaAs-based fiber optic probe immersed in the sample. Equation (7) estimates the heating efficiency of nanoparticles quantified in terms of the specific loss power (SLP), which determines the power dissipation per unit mass of magnetic material (in W/g) by using the following formula:

(7) $$SLP=c\frac{m_{f}}{m_{mag}}\frac{dT}{dt},$$

where *c* is the specific heat of the solvent, *m_f_* is the ferrofluids mass, *m_mag_* is the mass of magnetic nanoparticles, and *dT*/*dt* is the slope of the adiabatic straight line in the temperature versus time curve that is obtained by the hyperthermia measurements, following the modified law of cooling, in a rigorous standardized procedure [78], routinely implemented to eliminate non-magnetic heat exchanges.

**Figure 10:**
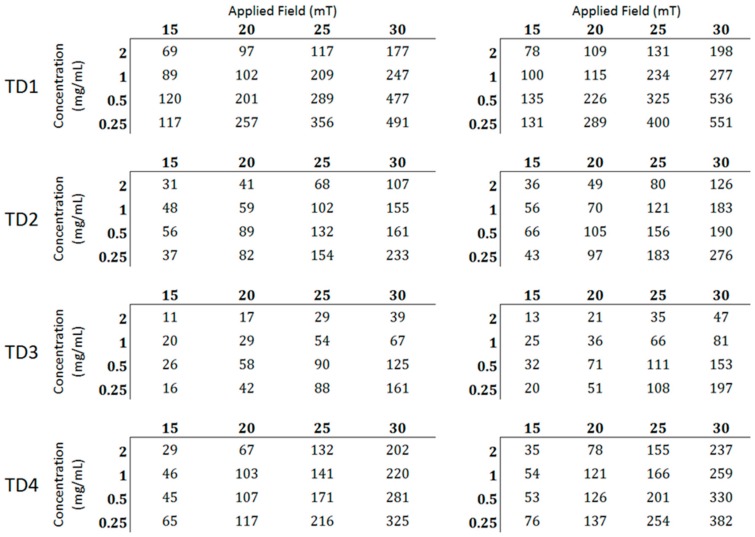
At the left tables the net specific loss power (SLP) values (W/g) per field and concentration are presented. At the right tables the values scaled to the metallic cores are presented.

Figure 10 shows the SLP values of TD1, TD2, TD3, and TD4. The values in the tables at the right side concern the SLP values scaled to the metallic core (W/g_Fe3O4_), namely the mass of the surfactants was deducted, while the left ones to the net SLP values (W/g). It becomes obvious that the decrease of the concentration of the colloidal leads to an increase of the SLP values in all samples, mainly at the higher field amplitudes (25 and 30 mT). Furthermore, as expected, the gradual increase of the field amplitude leads to a corresponding increase of SLP values, apparently because of the increase of the offered energy [42]. The decrease of SLP with the increase of concentration may be derived from the increased value of the field of dipolar interactions between the particles of the colloidal [79,80], which occurs mainly at high concentration colloidal.

**Figure 11:**
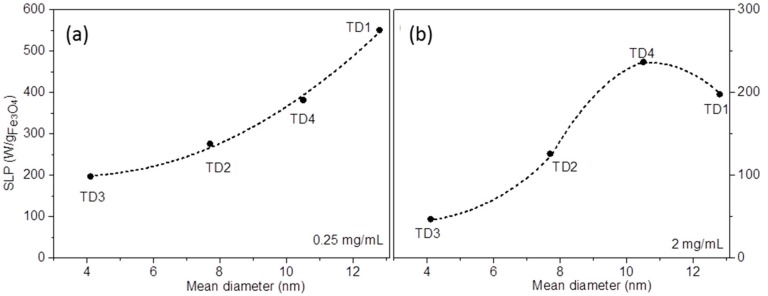
SLP values versus mean diameter of nanoparticles. The graphs refer to measurements that were carried out at concentrations (**a**) 0.25 mg/mL and (**b**) 2 mg/mL. At 0.25 mg/mL the graph is parabolic, while at 2 mg/mL a maximum value appears for TD4 (10.5 nm).

The maximum SLP values for each sample are 551, 382, 276, and 197 W/g_Fe3O4_ for TD1, TD4, TD2, and TD3, respectively. These values decrease with the decrease in the mean diameter of the nanoparticles. In other words, the SLP value is quite controllable from the polyol that is used for the synthetic procedure. Figure 11a shows the curve of SLP versus the mean diameter of the nanoparticles. The SLP values refer only to a concentration of 0.25 mg/mL and a field amplitude of 30 mT. This curve is parabolic with formula:

SLP = A + B·<d> + C·<d>^2^, (8)

where A, B, C are constants, and <d> is the mean diameter of the nanoparticles. Equation (8) is an empirical mathematical formula giving the trends of the increase and is used as guide to the eye. By fitting, the three constants were found to be A = 253.6, B = −30.2, and C = 4.1. This formula concerns only magnetite nanoparticles with mean diameters between 4 and 13 nm. Martinez-Boubeta et al. [80] found a similar parabolic dependence for Fe-MgO core-shell nanoparticles at a concentration of 0.5 mg/mL and 30 mT applied field, values close to ours. Generally, the main tendency that is found in literature about the SLP dependence from mean diameter follows a sharp increase until a maximum, while at larger diameters the SLP value decreases. In computational simulations for magnetite nanoparticles that have been derived from Phong et al. [81], the maximum SLP appears at 19 nm, whereas from the experimental results that have been obtained by Khandhar [82] et al. the maximum SLP value appears at 16 nm, for magnetite nanoparticles as well. In our experiments the diameter of the nanoparticles is smaller and, therefore, only the sharp increase of SLP appears. 

At higher concentrations this trend is not followed. Figure 11b shows the same curve at a concentration of 2 mg/mL. SLP increases gradually with diameter until a maximum at 10.5 nm, while the SLP decreases for larger nanoparticles. A similar trend was found by Bakoglidis et al. [5] for Fe_3_O_4_/γ-Fe_2_O_3_ nanoparticles, where at concentration 0.3 mg/mL and field amplitude 25 mT the SLP vs. mean diameter curve is similar to the curve of Figure 11b. However, in our results the same tendency appears at much higher concentration (2 mg/mL), whereas at 0.25 mg/mL, a value close to 0.3 mg/mL, the aforementioned parabolic curve occurs. The result of this differentiation may be due to the enhanced dipolar interactions between the nanoparticles of TD1. Particularly, as one can see in Equation (4), the increase of concentration leads to decrease of this distance and, consequently, to enhancement of dipole interactions. This enhancement causes a corresponding strengthening of stability against the magnetic field [5]. It should be mentioned that the decrease of SLP with the increase of concentration occurred in all samples, but the decrease in the case of TD1 is more intense probably because the magnetization of TD1 is the highest. As a result, at 2 mg/mL (Figure 11b), the curve is flagging at larger diameters, where the moments are increased. The concentration effect on SLP is not monotonous resulting in large variations among different authors in literature [83,84]. It seems that, up to a point the increase of the concentration magnetically hardens the MNPs either by ordered or disordered clustering [85]. It should be noted that the externally applied AC magnetic field should be strong enough to manipulate the MNPs solution, which at the same time has to maintain its colloidal stability; two reasons why SLP may attenuate above a critical concentration. A detailed discussion of the SLP dependencies on experimental parameters including particle size can be found in [78].

**Figure 12:**
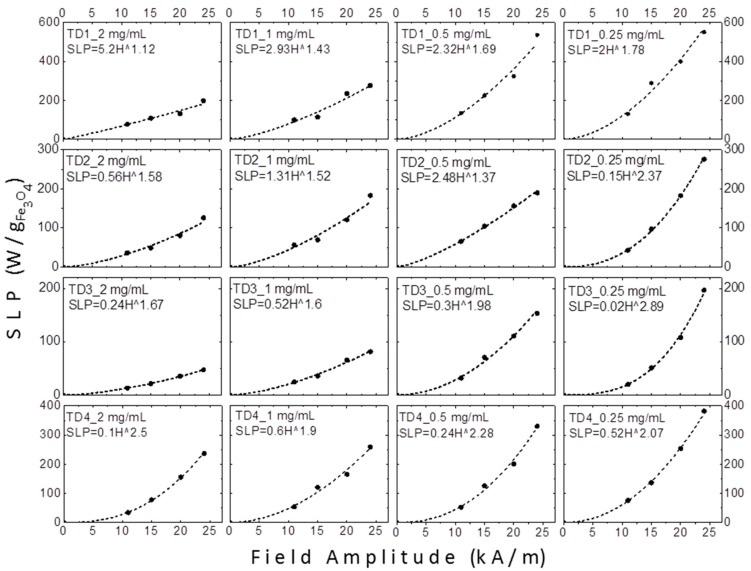
The SLP values of TD1–TD4 at four different concentrations (2, 1, 0.5, and 0.25 mg/mL) versus the amplitude of the applied field in kA/m. On each graph the formula which approximates the SLP as a function of the field amplitude is given.

Figure 12 shows the graphs of SLP values versus amplitude of applied field in kA/m. As mentioned above, at low fields the SLP could be proportional to H^2^ for superparamagnetic nanoparticles or H^3^ for thermally blocked nanoparticles [43]. When hysteresis losses and relaxation values coexist, the exponent gets intermediate values, such as 2.5 [45]. However, in samples TD1–TD4 a safe conclusion about the field amplitude dependence of SLP could not be extracted, since different exponents were calculated.

**Figure 13:**
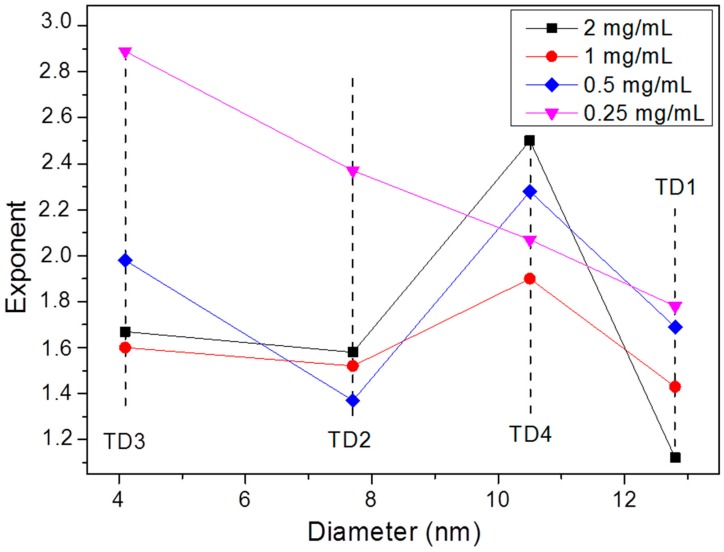
The exponent of field amplitude in the relation SLP~H^ct^ versus the diameter of the nanoparticles. At 0.25 mg/mL a clear decrease of the value of the exponent is observed with the increase of the diameter. At higher concentrations, where the dipolar interactions are enhanced, this order is disrupted. At TD4 (10.5 nm) the exponent receives the highest value, for 2, 1, and 0.5 mg/mL.

Carrey et al. [86] have found a relation between the field amplitude exponent and the radius of the nanoparticles, by fitting the hysteresis loops and by calculating theoretically their areas for several radius values of the nanoparticles. They found that the exponent for a nanoparticle with radius equal to 4 nm, namely with diameter equal to 8 nm, receives a value equal to 2. For larger nanoparticles, as the radius gradually increases, the exponent decreases, respectively, up to a radius of 9 nm, where the exponent receives a value of 0.6. For even larger nanoparticles with radius 10 or 12 nm, the exponent receives values 2 or 6, respectively [86]. Concerning our samples, TD1–TD4, factors like the colloidal concentration or the particle interactions enhance the uncertainty. Despite this, as shown in Figure 12 and Figure 13, at 0.25 mg/mL a gradual increase of the exponent with the decrease of the diameter of the nanoparticles is observed. At this concentration the dipolar interactions are weaker in comparison with the interactions at higher concentrations. The exponents are 1.78, 2.07, 2.37, and 2.89 for the samples with nanoparticles diameters equal to 12.8, 10.5, 7.7, and 4.4 nm, samples TD1, TD4, TD2, and TD3, respectively. Τhis pattern ceases at higher concentrations as the exponent always maximizes for sample TD4. Figure 13 shows the dependence of the exponent versus the diameter of nanoparticles; only at a concentration of 0.25 mg/mL the clear conclusion described above is extracted.

## 4. Conclusions

By both the thermal decomposition (TD) and the solvothermal method (SV), iron oxide nanoparticles were produced that consisted mainly of magnetite. The polyol process was found suitable for size control in both chemical approaches. The presence of a reducing agent (NaBH_4_) led to an increase in the size with the decrease of molecular weight of the polyol, while its absence led to the opposite result. Additionally, the high molecular weight polyols, such as PEG-8000, are suitable for structuring microspheres from nanoparticles. In this case the nanoparticles came closer and, as a result, the coercive field of the sample was enhanced because of dipolar and/or exchange interparticle interactions.

Since the size of the nanoparticles is controllable, the magnetic features and, therefore, the heating efficiency of the nanoparticles is controllable, as well. By using the appropriate polyol the SLP value reached a maximum of 551 W/g_Fe3O4_ for the TD sample that was synthesized with TrEG, while by using polyols with higher molecular mass, the SLP value decreased. The decrease in concentration led to SLP decrease for all samples, but this decrease was more intense for the sample with the highest magnetization. The cause of this attitude was the dipole interactions as well. Finally, a relation of the type SLP~H^ct^ between SLP and field amplitude was obtained. The exponent is directly related with the mean diameter of the nanoparticle. Hence, not only the SLP values could be controlled by the synthetic conditions and the polyol, but this exponent is completely controllable as well.

The polyol process in iron oxide nanostructuring besides the fact that is a facile and low-cost method, offers combined control in nanoparticle’s size, magnetic features, thermal ability, and the ability of hierarchical microstructuring from nanoparticles.

## Figures and Tables

**Table 1:** Synthetic protocols of the thermal decomposition samples TD1–TD4.

Sample	Initial Components in Flask	Hot Injection (100 °C)	Final Temperature (°C)
TD1	5 mmol Fe(acac)_3_ 300 mmol TrEG (40 mL) 5 mmol NaBH_4_	-	260
TD2	2.5 mmol Fe(acac)_3_ 20 mmol PEG-1000 (20 g)	2.5 mmol NaBH_4_	260
TD3	2.5 mmol PEG-8000 (20 g)	2.5 mmol Fe(acac)_3_ 2.5 mmol NaBH_4_	260
TD4	2.5 mmol Fe(acac)_3_ 150 mmol TrEG (20 mL) 2.5 mmol N_2_H_4_·H_2_O	-	260

**Table 2:** Synthetic parameters of the solvothermally produced samples, SV5, SV6, and SV7.

Sample	Reactants	Temperature (°C)
SV5	1.5 mmol Fe(acac)_3_ 75 mmol TrEG (10 mL)	200
SV6	1.25 mmol Fe(acac)_3_ 10 mmol PEG-1000 (10 g)	200
SV7	1.25 mmol Fe(acac)_3_ 1.25 mmol PEG-8000 (10 g)	200

**Table 3:** The mean size values of the nanoparticles of samples TD1–TD4, as calculated by the TEM images, and the mean size values of the crystallites of all samples as calculated by Scherrer’s formula.

TD Samples	Nanoparticles’ Mean Size (nm)	Crystallites’ Mean Size (nm)	SV Samples	Crystallites’ Mean Size (nm)
TD1 (TrEG)	12.8 (±3.3)	11.8	SV5 (TrEG)	8.6
TD2 (PEG-1000)	7.7 (±1.6)	8.1	SV6 (PEG-1000)	13.7
TD3 (PEG-8000)	4.1 (±1.0)	4	SV7 (PEG-8000)	18.7
TD4 (TrEG/hydrazine)	10.5 (±2.2)	11.9	-	-

**Table 4:** The magnetic features of samples TD1–TD4. M_s_ represents the scaled saturation magnetization, M_r_ the scaled remanence magnetization, and H_C_ coercivity. The M_s_ values decrease gradually with the mean size.

Sample	M_s_ (Am^2^/kg_Fe3O4_)	M_r_ (Am^2^/kg_Fe3O4_)	M_r_/M_s_ (%)	H_c_ (mT)	Mean Size (nm) by TEM
TD1	73.7	34.2	46.3	17.8	12.8
TD2	40.2	11.3	28.2	12.2	7.70
TD3	12.6	0.9	6.80	18.4	4.10
TD4	66.0	6.20	9.40	14.0	10.5

**Table 5:** Magnetic features of samples SV5, SV6, and SV7.

Sample	M_s_ (Am^2^/kg)	M_r_ (Am^2^/kg)	M_r_/M_s_ (%)	H_c_ (mT)	Crystallite Size (nm) by Scherrer’s Formula
SV5	64.1	16.2	25.3	17.6	8.60
SV6	53.3	16.1	30.2	17.8	13.7
SV7	46.3	11.0	23.8	18.5	18.7

**Table 6:** Comparison of saturation magnetization values of TD1-SV5, TD2-SV6, and TD3-SV7 pairs. These values are not justified for the metallic core mass. The molar ratio between the precursor and the surfactant remained constant in any samples’ pair.

Sample	M_s_ (Am^2^/kg)	Precursor mol/ Surfactant mol	Sample	M_s_ (Am^2^/kg)	Precursor mol/Surfactant mol
TD1	65.6	5: 300	SV5	64.1	1.25: 75
TD2	34	2.5: 20	SV6	53.3	1.25: 10
TD3	10.3	2.5: 2.5	SV7	46.3	1.25:1.25
